# Integrated care for chronic respiratory disease: a narrative review

**DOI:** 10.1183/16000617.0192-2025

**Published:** 2026-04-13

**Authors:** Michael C. Steiner, Louise Ryan, Irene Valero-Sanchez, Onyeka Umerah, Thomas J.C. Ward

**Affiliations:** 1 Department of Respiratory Sciences, University of Leicester, Glenfield Hospital, Leicester, UK; 2 NIHR Leicester Biomedical Research Centre, Glenfield Hospital, Leicester, UK; 3 NHS Leicester, Leicestershire and Rutland Integrated Care Board, Leicester, UK; 4 Department of Thoracic Medicine, University Hospitals of Leicester, Glenfield Hospital, Leicester, UK

## Abstract

Integration of healthcare services for people with chronic respiratory disease (CRD) is proposed as a means to improve the delivery of evidence-based therapies, thereby improving outcomes, experience of care and reducing health inequalities. In this narrative review we define categories of integration interventions, present a structured assessment of the evidence supporting both horizontal and vertical integrated care models and case-finding in CRD, and describe the implications for the implementation and scaling of integrated care programmes in clinical practice. Our findings suggest better coordination of the provision of evidence-based interventions through horizontal integration can improve clinical outcomes in CRDs, including reducing episodes of unscheduled care. Vertical integration provided by different organisational units in healthcare systems can enhance delivery of evidence-based interventions but impact on longer-term outcomes is less clear. The available evidence, whilst providing support for integration, does not conclusively support a specific model of care or organisational structure. Careful attention is required to define components of integrated care interventions. Study duration and choice of outcomes are key to evaluating clinical and cost effectiveness in future trials.

## Shareable abstract

**Access to evidence-based therapies for chronic respiratory disease requires improvement. The available evidence suggests better integration of services can improve access and outcomes but does not conclusively support a specific model of care.** https://bit.ly/4sw0Sh7

## Introduction

Chronic respiratory diseases (CRDs), most notably chronic obstructive pulmonary disease (COPD) and asthma, are prevalent long-term conditions (LTCs) that impose a significant burden on patients, their families and healthcare systems globally [1]. Despite ample evidence that many of these conditions are preventable and responsive to timely, evidence-based interventions, delays in diagnosis and fragmented delivery of care remain persistent challenges. The burden is magnified in underserved populations, where higher rates of smoking, environmental exposures and barriers to accessing healthcare contribute to steeper gradients of disease prevalence and outcomes [2].

Scientific development is delivering an increasing number of effective interventions for CRDs (for example biologic drug therapies) further increasing the complexity of healthcare delivery. However, despite the availability of highly clinically and cost-effective interventions, including smoking cessation, pulmonary rehabilitation (PR), vaccination and pharmacotherapy, a significant proportion of patients still do not to receive optimal basic care [3–5]. Moreover, there is compelling evidence for an increased prevalence of multiple long-term conditions (MLTCs), including mental health problems, in people with CRDs, which further exacerbates the burden imposed by the condition and healthcare requirements for individual patients.

Re-organisation of care through better integration of health and care services has been proposed as a means to enhance the clinical and cost effectiveness of healthcare provision for people with LTCs, improve patient experience of care and reduce health inequalities.

In this review we:

1) Set out definitions of the terminology used when integrated care programmes are described.
2) Critically assess the evidence from the available recent scientific literature testing the effectiveness of integrated care models for patients with CRD. Because of the wide definition of integrated care in the literature, we placed the greatest emphasis on evidence from randomised controlled trials (RCTs) and prospective controlled studies of care models for the diagnosis and management of CRDs.
3) Draw conclusions regarding effective integration models and discuss the implications for scaling these approaches in real-life clinical practice, with a view towards improving long-term care models for chronic diseases.

## Definitions used

The importance of service coordination in a complex healthcare landscape is reflected in the development of government driven healthcare reform; for example, the establishment of Accountable Care Organisations in the USA and the UK Health and Care Act 2022, bringing about the formation of integrated care systems. Inevitably, the definition of “integration” will vary internationally but the systematic assessment of the scientific evidence in this field is also hampered by:

- Variations in terminology: the terms “integrated care”, “chronic disease management”, “care coordination” and “integrated disease management” are used interchangeably across studies, complicating the aggregation of evidence.
- Diverse reporting formats: many service innovations and quality improvement initiatives have been reported only as abstracts or on society platforms, making systematic evaluation difficult.
- Limited controlled studies: the number of rigorously controlled experimental studies is limited, necessitating the inclusion of observational and pragmatic trial designs.

Definitions of integration have varied according to the perspective of healthcare providers, users and policy makers (readers are directed to previous reviews [6–8]). In a report in 2017 from the European Commission (“BLOCKS”) [9], integrated care is defined as “…initiatives seeking to improve outcomes of care by overcoming issues of fragmentation through linkage or co-ordination of services of providers along the continuum of care”. In the UK, a joint position paper from the British Thoracic Society and the Primary Care Respiratory Society [10] proposes that integrated respiratory care should be “patient-centred, proactive, and coordinated by multi-professional teams working across organisational boundaries”. For the purposes of this review (and outlined in the BLOCKS report) [9], we distinguish between horizontal and vertical integration as follows.

### Horizontal integration

This refers to the coordination of services at the same level of care. Examples include linking general practice with community-based services or co-locating mental and physical healthcare. Many integrated disease management programmes, which incorporate elements such as smoking cessation, exercise and self-management education, fall into this category. Incorporating the care of multiple different LTCs in an individual would also fall into this category. Taking a holistic approach to the needs of the patient, including social and mental health needs, is a key aspect of horizontal integration but may also require vertical integration between healthcare organisations.

### Vertical integration

Vertical integration involves bridging different organisational units of the healthcare system; for example, bringing together primary care, secondary care and specialist services under a unified management structure. This approach is typified by initiatives that embed specialist expertise into primary care settings or outreach hospital services into the community, thereby ensuring more seamless diagnostic and management processes. Similarly, integrating health and social care may fall under this category if provision is delivered by separate organisational structures.

**FIGURE 1:**
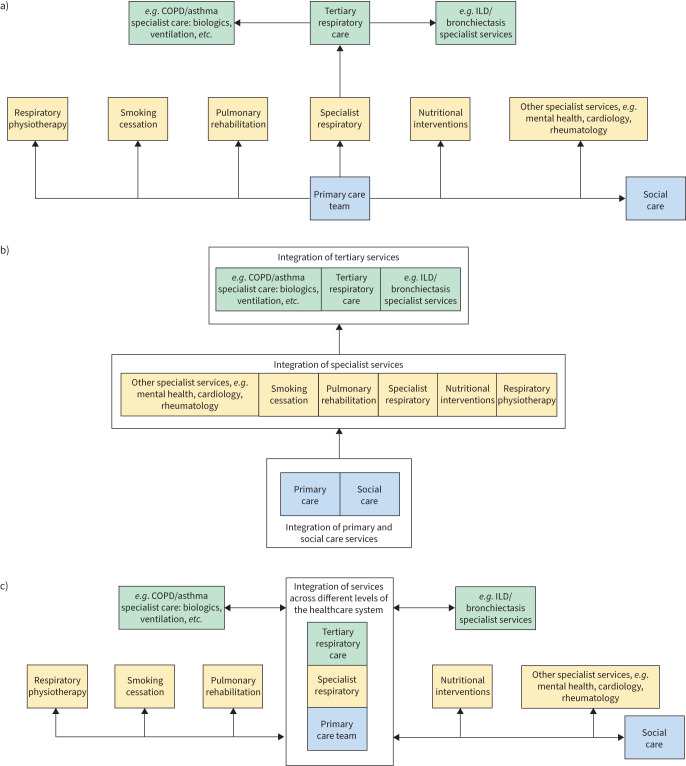
Models of horizontal and vertical integration. a) The traditional model of care. Referral and discharge between primary and secondary care and between discrete services for chronic respiratory diseases occurs at the discretion of the referrer and receiving service, and are reliant on patients expressing their needs and referrers recognising their requirements. b) Horizontal integration. Services/interventions at the same “level” of care are coordinated and accessed by patient populations in a systemised and organised manner. This allows similar services to be clustered together to improve efficiency and outcomes for patients. c) Vertical Integration. Health services or organisations that provide care at different “levels” are brought together to deliver coordinated care rather than requiring transitions between these organisations. In some examples of integrated care interventions, elements of horizontal and vertical integration overlap or are combined. ILD: interstitial lung disease.

In summarising studies of these categories of integration (see figure 1), we recognise the boundaries between them are not absolute and some studies incorporated elements of both in their design.

## Search methodology

We undertook a narrative review informed by a literature search supplemented by the Cochrane systematic review of integrated disease management in COPD [11]. A search of the literature was undertaken (by an experienced clinical librarian, see acknowledgements) of the Medline, Embase and Emcare databases (*via* Ovid) to 18 November 2024. Appropriate subject headings and keywords were used for COPD, asthma and managed care programmes. Highly sensitive search strategies were used to identify RCTs [12]. We took a hierarchical approach, placing greater emphasis on RCTs and prospective studies over retrospective analyses or uncontrolled service evaluation reports. We focused on studies of adult and paediatric CRD integrated care after 2020 but included earlier studies where these were relevant and pivotal. Given the importance of timely and accurate diagnosis of CRDs, we included case-finding studies where the impact on outcomes and care processes was measured as well as diagnostic capture rates. A summary of the literature identified and assessed is provided in the online supplemental table.

## Integrated case-finding pathways for respiratory disease

There is evidence that diagnosis of conditions such as COPD and asthma is often delayed or inaccurate. For example, in the SUMMIT study, diagnostic spirometry was undertaken in participants undergoing computed tomography lung cancer screening. Where symptoms of COPD were present (using a standardised questionnaire) and no prior diagnosis of COPD had been made, case identification rates were in the region of 20% [13]. Therefore, structured and proactive programmes of case identification (case-finding) may therefore be beneficial [14, 15].

Using a telephone survey methodology, individuals in Canada were screened for respiratory symptoms and if these were present validated asthma and COPD symptom scores were undertaken. Of those with symptoms but without a prior diagnosis of asthma or COPD, around 20% were found to have either asthma or COPD based on diagnostic spirometry [16]. In a subsequent Canadian study, individuals identified to have asthma or COPD using this case-finding methodology were randomly allocated to receive standard primary care or a specialist intervention (review by a pulmonologist and a COPD-asthma educator) [17]. Primary care healthcare utilisation was significantly lower and health status was significantly improved in the specialist intervention arm.

Other trials have evaluated the effectiveness of proactive support for primary healthcare teams in diagnosing CRDs. In a cluster RCT undertaken in the UK, general practitioner (GP) practices were randomised to a targeted case-finding intervention (a screening questionnaire followed by spirometry if indicated) compared to usual care. The targeted case-finding group was further randomly allocated to do this opportunistically (when the patient attended the practice) or actively (at-risk individuals identified from practice databases and mailed the questionnaire). The yield of COPD diagnoses in the intervention arm was greater than in the control arm (4% *versus* 1%) and was also greater in the active case-finding group. In a health economic analysis, these case-finding approaches were found to be cost-effective. In a follow-up study (up to 4 years) the diagnosis of COPD did result in the provision of evidence-based COPD interventions, but this was incomplete and there was no significant difference in hospitalisation or mortality rates between the groups [18]. In a cohort study using the same screening questionnaire, practice databases in the South of England were scrutinised and patients at risk of COPD were invited to attend for diagnostic spirometry [19]. Rates of identification of COPD in those that attended were in the region of 25%, but at 12 months only around 10% of these had received a diagnostic label of COPD on their primary care record suggesting that opportunities for therapeutic intervention may have been missed.

In the DISCO study in France, the value of the routine use of Global Initiative for Chronic Obstructive Lung Disease (GOLD) questions with or without coordinated provision of spirometry for adults over 40 years old was tested in a cluster RCT using a 2×2 factorial design [20]. Combinations of usual care, use of GOLD screening questionnaires [21] and/or the support of a dedicated healthcare worker to coordinate spirometry were compared. No cases of COPD were identified in the control group compared with 1% of cases diagnosed using the case-finding approach. Rates of COPD identification were higher where the coordinator was deployed, although this might be explained by the lack of availability of primary care spirometry in France requiring referral to a specialist centre for diagnosis.

## Integrated care programmes for respiratory disease

### Horizontal integration

In an updated Cochrane systematic review, Poot *et al.* [11] summarised 52 trials of “integrated disease management” interventions, taking 26 studies included in a first review undertaken in 2013 [22] and adding a further 26 studies published subsequently. Care interventions were identified based on a previously defined set of criteria (taxonomy) of which at least two were required to be included as components to meet the definition of integrated disease management and therefore be included. Overall improvements in health status, exercise capacity, hospital admissions and bed days were observed. There was considerable heterogeneity in these outcomes and indeed in the content of included studies. Many studies describe horizontal integration of components of treatment that are already frequently co-located in order to address the multifaceted needs of people suffering with CRDs. In some, elements of vertical integration were addressed through facilitated access to specialist advice [23–25].

In many health systems, the horizontal integration of components of evidence-based care for people with CRDs are bundled into PR programmes. In line with this, the COPDnet trial in the Netherlands enrolled people with COPD to an observational study of an integrated care model focusing on nonpharmacological therapies. They observed that improvements in health status following the intervention were largely mediated through attendance at PR [26]. PR itself, whilst often deployed as a single “intervention”, comprises a range of components addressing a variety of patient needs and as such is a form of horizontal integration [4, 27, 28].

Koff *et al.* [29] tested the effectiveness of a structured, proactive model of COPD care that integrated a patient education programme with home monitoring and support for communication with the healthcare team. Improvements in health status and a reduction in unscheduled episodes of care were observed in the active treatment group. Liang *et al.* [30] reported the findings of a cluster RCT in which a comprehensive package of COPD disease management components (home medication review by a pharmacist, home PR and support for smoking cessation) were provided to GP practices in the active intervention arm. The trial did not show effects on the primary intention-to-treat outcome of health-related quality of life. This might have been explained by low uptake of the intervention as positive effects were observed in a per-protocol analysis.

COPD frequently co-exists with MTLCs. This is an increasingly burdensome problem for patients and healthcare services. Managing polypharmacy, increased complexity of access to specialist services (which usually focus on a single organ speciality) and constraints on capacity and capability in primary and community care services in managing complexity are rising challenges [31]. A detailed review of generic trials of integrated programmes for MLTCs is beyond the scope of the current review but has been summarised in policy documents and Cochrane reviews elsewhere [32, 33]. A systematic review of integrated care interventions for people with chronic conditions suggested there may be benefit from individual case management and the deployment of multiple elements of the chronic care model (CCM) [34, 35]. This is supported by an earlier systematic review summarising the impact of elements of the CCM on outcomes in COPD which suggested that two or more components needed to be deployed for the intervention to be effective [36]. Studies applying an intervention in patients with different LTCs (as opposed to managing complex co-existent MLTCs) suggest many therapies, such as rehabilitation or digital support platforms, can be generically offered in a disease agnostic manner [37–39]. It is likely these would benefit patients with MTLCs but controlled trials of the horizontal integration of such programmes focused on CRDs specifically are lacking.

### Vertical integration

In many health systems, vertical integration has been implemented and studied as part of service or quality improvement. For example, in respiratory disease in the UK, reports by Hull *et al.* [40] and Patel *et al.* [41] showed improvements in care delivery and coordination between primary and secondary care, which appeared to translate into reduced emergency care utilisation, although these were not controlled studies and comparators were historical admission rates. In the MISSION ABC study, a 12-month prospective observational study in the UK, specialist-led disease management reviews for people with asthma or COPD were delivered in primary care settings, complemented by digital monitoring tools [42]. Patients were selected from primary care COPD and asthma registries or those with coded episodes of unexplained breathlessness. The reviews resulted in diagnostic change in 17% of participants and treatment changes in 57%. The initiative resulted in increased clinic expenses and drug costs, but these were offset by a reduction in episodes of unscheduled care resulting in an overall cost reduction.

More recently, controlled studies of vertical integration of respiratory services have been reported. Patel *et al.* [43] conducted a cluster RCT in which GP practices in Birmingham, UK were allocated to receive support for COPD reviews from a specialist team (including a respiratory physician and allied professions based in the local hospital) or usual care from their primary care physician. Practices receiving the additional specialist support delivered better adherence to guideline-based elements of care (smoking cessation support, vaccination, offer of PR and medicines management). Much of this improvement was driven by higher referral rates to PR in the intervention arm. Health status improved in the intervention arm but counterintuitively there were also higher rates of COPD related hospital admissions in this group, although this may be due to differences in methods of collecting admission data between groups [44]. It is also notable that the study was conducted in the context of a local healthcare initiative whereby virtual reviews of COPD care were available to all primary care providers enrolled to the study and therefore the applicability to other healthcare landscapes is uncertain. Saini *et al.* [45] studied the impact of a community-based, consultant-led COPD service in the North West of England providing a consultant-led community COPD clinic, a nurse-led rapid response and early supported discharge service, a community home oxygen service, integrated palliative care, and counselling services. In a longitudinal matched control analysis, they demonstrated a small but nonsignificant reduction in emergency admissions following the introduction of the service but did not collect data on wider health outcomes.

Wolfe *et al.* [46] reported the findings of a cluster RCT of a paediatric integrated care programme (the Children and Young People's health partnership (CYPHP)) focusing on children with “tracer” conditions (asthma, eczema and constipation). 70 participating GP practices (responsible for the care of just under 100 000 children) were allocated to clusters and randomised to an offer of access to the CYPHP (comprising practice-based specialist paediatric clinics and nurse-led early intervention clinics for at-risk children) in addition to “enhanced usual care”, which was also offered to control practices. This comprised decision support and guidelines, a hotline between GPs and paediatricians together with self-management and school-based support for patients managed by these practices. No difference between the groups in the primary outcome (episodes of unscheduled emergency hospital care) was observed and, similarly, paediatric quality of life scores were unaffected. However, improvements in the quality of care for children with asthma were noted, including better assessment of asthma control and provision of asthma self-management plans. At 12 months, a health economic analysis concluded that the programme was not cost-effective, although trends to improved cost-effectiveness over time were reported [47]. The authors advance a number of explanations for the negative findings of the trial, including slow uptake and disruption due to the COVID-19 pandemic. The authors acknowledged that the intervention may not have been effective in modifying healthcare use in its target population, although this contrasts with earlier reports from an observational study of an integrated care intervention (principally aimed at supporting patients/parents and primary care) in Australia suggesting rates of unscheduled care were reduced (albeit in a nonrandomised design) [48].

Other studies have tested vertical integration in the form of specialist support for primary care teams rather than providing direct specialist delivery of care in partnership with other care sectors. Kruis *et al.* [49] performed a cluster RCT (the RECODE trial) testing the effectiveness of a package of support and training for primary care teams managing patients with COPD. Overall, there was no impact on health-related quality of life or other patient-reported outcomes. However, an increase in the proportion of patients being more physically active increased, as did a measure of the coordination of healthcare in the active intervention group. In a cluster RCT, Broese *et al.* [50] tested the impact of offering a training package to healthcare teams treating people with COPD; in this case, the provision of palliative care for patients. No impact on health-related quality of life or other patient-related outcome measures was observed, although the trial was truncated by the COVID-19 pandemic and may have been underpowered for these outcomes. However, unscheduled hospital and intensive care unit visits were reduced, potentially mediated by more proactive discussions of advance plans and palliative care needs in the active intervention group.

## Digital technologies to support integration

Digital technologies have the potential to be useful tools in integrated respiratory health, allowing teams to communicate better and allowing routine identification of patients with unmet needs. There has recently been an interest in the use of artificial intelligence (AI) to improve healthcare efficiency, enhance risk prediction and identify therapeutic opportunity. There is limited evidence for the benefit of such an approach over usual care. For example, early prediction of COPD exacerbations may allow targeting of community resources and avoidance of hospital admissions. However, to date, machine learning models have not consistently performed better than traditional risk scores [51].

Many integrated care models use digital technologies to support the delivery of their services. For example, the MISSION ABC study included digital platforms for monitoring respiratory symptoms and to support self-management [42]. Digital platforms have the potential to allow closer monitoring of patients at home and lower-cost delivery of education and self-management resources. However, digital support platforms may be expensive [3] and might worsen health inequalities as poorer digital literacy is associated with greater socioeconomic deprivation [52].

Digital technologies have been used to support the development of respiratory virtual wards, which have increasingly been used since the COVID-19 pandemic to support patients with respiratory disease who would otherwise be hospitalised. It is beyond the scope of this article to provide a comprehensive review of respiratory virtual wards, which has been published elsewhere [53]. However, although they are likely to be a safe alternative to hospital care for many patients, there is limited evidence of the impact of virtual wards on hospital admissions or healthcare costs.

## Discussion and implications for future provision of integrated care for CRDs

Traditional models of healthcare provision, distinguishing primary and/or community care from specialist/secondary care, have developed historically to meet the needs of discrete episodes of care, such as elective surgery or acute illness followed by recovery. Payment arrangements often follow these episodes and may entrench traditional models through financial incentives. These arrangements are less suited to the needs of people with LTCs, including those with CRDs, where ongoing regular review, recurrent treatment for periods of acute decompensation and management of other aspects of health, for example mental health, are required. The integration of healthcare to bring these sectors together, centred around the needs of the patient (vertical integration), may therefore yield improvements in clinical outcomes and patient experience of care. Differential impacts of integrated care interventions based on socioeconomic or other inequalities are infrequently reported. Saini *et al.* [45] reported better outcomes in populations with “medium” levels of socioeconomic deprivation (compared with lower or higher deprivation groups) and in men. In the SUMMIT study, Tisi *et al.* [13] reported higher rates of previously undiagnosed COPD in ethnic minority populations and in men. The impact of integrated care interventions on the scale of health inequality has not been reported and indeed might be difficult to demonstrate given the known broader social determinants of health.

There is substantial heterogeneity of trial design, outcome measures and composition of interventions in studies of integrated care for CRDs. As a result, there is a lack of clear consensus on how such models of care should be implemented. Inevitably, interpretation of the available evidence will depend on the healthcare setting or system where change is proposed. However, there are some important themes arising from the available evidence that can inform health service redesign, identify barriers and enablers to successful integration and guide future research.

### Themes arising from the scientific evidence

1) Proactive case-finding can be an effective means to promote timely and accurate diagnosis in people with CRDs. However, evidence suggests that, for outcomes to be improved, case-finding needs to be linked to integrated disease management and support for primary care. Overall diagnosis rates in many studies might be considered to be low (less than 10%), but rates of undertaking spirometry were high and the value of diagnostic “rule-out” (for example, reduction in inappropriate prescribing) was not captured and might be substantial. Enriching case-finding programmes by targeting cohorts at particular risk (for example, embedding them in lung cancer screening programmes where emphysema is identified) may yield higher capture rates.
2) Horizontal co-location of elements of evidence based respiratory care is likely to improve outcomes and reduce healthcare utilisation where this is suboptimally provided. In many systems, this can be provided as part of PR but referral and uptake rates are often low [4, 54]. Measures to enhance PR uptake rates and better organisation of care for patients who are unable or unwilling to attend PR may be an effective means to enhance horizontal integration [55]. Evidence is lacking on effectiveness of horizontal integration for people suffering with CRD and MTLCs despite many of the highest value interventions being common across many such LTCs (*e.g.* rehabilitation, secondary prevention drug therapy, vaccination, tobacco dependency, palliative care). Trials of service redesign in this area are urgently needed, given the rising prevalence of MLTCs and the costs ensuing for healthcare systems.
3) Evidence from studies of vertical integration in CRDs suggests that processes of care (the delivery of items of evidence- or guideline-based care) can be increased through specialist support for primary care. Such support will be more effective if directly involved in care delivery. Training support alone may be insufficient to sustain this, especially in settings where there is significant stress on primary care services. Impacts on patient-reported measures such as quality of life or healthcare utilisation and cost-effectiveness are unclear and may require significantly longer durations of implementation and outcome assessment to evaluate.

### Challenges around the implementation of integrated care programmes

#### 1) Barriers and enablers to integration

 Integrated care is an exercise in bringing together and harmonising care provided to patients by different clinical teams and health providers. Many studies in this field conclude that active “buy-in” from relevant stakeholders and partners is a prerequisite for sustained success. This is supported by qualitative and survey data where this is undertaken for people with CRDs [56, 57]. Ensuring such commitment is harnessed at the start of integration programmes and aligning both programme expectations and aims across partners appear crucial. Ensuring financial flows incentivise cooperation rather than fragmentation may be important in underpinning this commitment, especially in the landscape of constrained funding that exists in many health economies.
  Digital technologies have a role to play, especially in facilitating information sharing and decision support between clinical teams. AI enhancements may add value to these processes, but more evidence is needed on clinical and cost-effectiveness. Such innovations have the potential to increase the efficiency of integrated programmes by facilitating a population health management approach to target interventions to populations of greatest need and therapeutic opportunity.

#### 2) Measuring outcomes

 The choice of metrics to measure success needs to be carefully considered and expectations need to be realistic. Many trials of integrated care programmes have struggled to demonstrate improvement in clinical outcomes in the short duration of observation involved in most studies. A consistent finding from many studies, however, is an improvement in the delivery of guideline-based therapies. These therapies are mandated in guidelines because clinical effectiveness has been demonstrated and therefore re-proving such effectiveness is not necessary. Indeed, doing so in the pragmatic setting of an integrated care programme may be unrealistic because of greater variations in population characteristics, insufficient sample sizes, *etc*.
  Cost-effectiveness and healthcare value need careful and rigorous evaluation. Many programmes are implemented with the objective of cost containment, but reductions in use of acute healthcare may be offset by increases in long-term treatment costs. Case-finding programmes may front-load costs by treating otherwise undiagnosed patients, although there may be savings through better disease control later in the natural history of the condition and a reduction in unnecessary prescribing for those without disease. The patient experience of care has not been consistently captured, but may be significantly improved through streamlining care processes and better communication between professionals.

## Conclusions

There is wide consensus that models of healthcare delivery for people with LTCs require reform and this clearly applies to CRDs. The available evidence, whilst providing support for integration of services in providing earlier diagnosis and more effective delivery of guideline-based care for people with CRDs, does not conclusively support a specific model of care or organisational structure. Careful attention to stakeholder buy-in, the judicious choice of outcome metrics and the implementation of appropriate financial incentives will be key to success. If effective, however, integrating healthcare for people with CRDs has the potential to transform health, wellbeing and the experience of care for patients, and the cost-effectiveness of service delivery.

## Supplementary material

**Please note:** supplementary material is not edited by the Editorial Office, and is uploaded as it has been supplied by the author. Supplementary table 1 ERR-0192-2025.SUPPLEMENT
