# Supplementary material for: Integrated care for chronic respiratory disease: a narrative review
Source: Eur Respir Rev. 2026 Apr 13;35(180):250192. doi: 10.1183/16000617.0192-2025 (PMC13169065; doi:10.1183/16000617.0192-2025)
Supplement: Supplementary file 1 [file ERR-0192-2025.SUPPLEMENT.pdf]

Table 1: Selected integrated care trials in respiratory disease

	Design	Country	Population	Intervention	Outcome	Conclusion
**Case finding**						
Tisi, 2022 (SUMMIT)	Single arm trial	UK	Current or former smokers aged 55-77 years old attending a lung cancer screening programme	Respiratory consultation and spirometry	19.7% met the criteria for undiagnosed COPD (symptoms, no previous COPD diagnosis and airflow obstruction)	High rates of undiagnosed COPD found. those undiagnosed were more likely to be male, currently smoking and from an ethnic minority group..
Aaron, 2024	Case finding followed by randomisation to early diagnosis and management or usual care	Canada	Adults over 18 years old with landline or mobile phone numbers within 90 minutes of 17 sites in Canada	Two step case finding – automated telephone call followed by screening questionnaire and if positive, pre and post bronchodilator spirometry. If undiagnosed asthma or COPD found – randomised to review by pulmonologist and an asthma–COPD educator or their GP (usual care)	595 new cases of asthma or COPD from 1,182,406 people receiving phone calls of which 38,353 had respiratory symptoms. Lower annualised rate of participant-initiated health care utilization for respiratory illness, better quality of life and greater FEV_1 improvement in the intervention group	Early diagnosis and intervention improves outcomes but very labour intensive and difficult to replicate in other health settings.
Adab, 2021 (BLISS)	Cluster RCT	UK	People who have smoked aged 40–79 years without a previous chronic obstructive	Symptom screening questionnaire, administered opportunistically or additionally by post, followed by diagnostic post-bronchodilator spirometry compared to usual care.	4% new COPD cases at 1 year in the intervention group compared to 1% in the usual care group. No difference in hospital admissions or mortality at	Intensive case finding identified new cases of COPD but uptake of evidence based COPD interventions was low and did not improve

			pulmonary disease diagnosis from 54 general practices		4 years.	outcomes.
Ray, 2021	Single arm trial	UK	Individuals identified as at risk for COPD using the TargetCOPD score applied electronically to the primary care routine electronic medical record	A diagnostic assessment at their GP practice by a specialist outreach nursing team with feedback to practices	26% of the 288 patient enrolled met spirometric criteria for COPD. However, of these only 11% had a diagnostic label of COPD in their primary care record. This constituted 0.38% of the total patient population, as compared with 0.31% in matched control practices	Case finding can identify undiagnosed COPD but without integration with primary care, does not automatically lead to a change in care
Chapron, 2023 (DISCO)	Cluster RCT	France	Patients aged over 40 in participating GP practices	GP practices randomised to delivery of a screening questionnaire, use of a COPD care co-ordinator, both or usual care	Of 3,162 patients enrolled, a new COPD diagnosis was made in 24 (0.8%)	Low diagnosis rate in an unselected population and no data on whether outcomes improved
**Horizontal integration**						
Koolen, 2020 (COPDnet)	Single arm trial	The Netherlands	Confirmed diagnosis of COPD referred to secondary care	Individualised specialist assessment focusing on identification and treatment of treatable traits. Focus on non-pharmacological interventions including pulmonary rehabilitation referral	402 patients enrolled with outcome data available for 154. Improvement in health-related quality of life driven by those attending pulmonary rehabilitation	Horizontal integration improves quality of life in COPD which appears to be mainly driven by increased pulmonary rehabilitation referrals.

Koff, 2021	Quasi-randomised controlled trial	USA	Severe COPD or COPD with a recent exacerbation recruited from primary and secondary care	A remote telehealth programme consisting of: 1) COPD education, 2) exacerbation education, 3) direct communication with study coordinators, and 4) remote home monitoring	511 individuals enrolled. Improvement in symptoms, health related quality of life and exercise performance. No effect on mortality.	An integrated home based programme focused around education and can improve patient related outcomes.
Liang, 2019	Cluster RCT	Australia	COPD or current/ex-smoker ≥40 years old	Smoking cessation support, a home medicines review and home based pulmonary rehabilitation under the supervision of their GP	272 individuals randomised. 31% in the intervention group completed the home medicines review and home pulmonary rehabilitation. Improvement in health related quality of life only in the pre protocol analysis but not the intention to treat. No improvement in symptoms, smoking abstinence or lung function.	The home based intervention was ineffective, possibly due to low uptake.
**Vertical integration**						
Hull, 2014	Quality improvement project	UK	Spirometric evidence of COPD	Focused on regular patient review including monitoring, management of co-morbidities, reviewing medicine use, and encouraging take-up of non-pharmacological interventions. Coordinator support	Increase in completed care plans, pulmonary rehabilitation referrals and flu vaccination rates following the intervention compared to baseline	Investment into collaborative working resulted in care provision in COPD

				for recall and management of non-attenders. Specialist support for spirometry training, pulmonary rehabilitation and a hospital admission avoidance service involving a same-day home assessment by respiratory nurse specialists.	levels	
Patel, 2016	Quality improvement project	UK	COPD	Developed a team of specialist respiratory nurses, physiotherapists, a pharmacist, a smoking cessation adviser, an integrated respiratory consultant and two GP leads. Weekly virtual clinics. Included accurate diagnosis, acute management of respiratory patients in hospital; supported discharge; COPD discharge bundle; caseload management of patients with complex breathlessness; a single point of referral and optimal respiratory prescribing.	Admissions for COPD reduced by 8% and length of stay reduced by 17%. Pulmonary rehabilitation referrals increased by over 50%.	Multidisciplinary working can reduce emergency care for COPD and increase uptake of pulmonary rehabilitation.
Heiden, 2024 (MISSION ABC)	Single arm trial	UK	Adults (≥16 years) with asthma, undifferentiated breathlessness and COPD	Specialist respiratory clinics in primary care with spirometry, oscillometry, FeNO and breathing control and inhaler technique education. Discussion at a specialist MDT and onward referral or investigation or discharge as required. Those at risk of exacerbations offered 3 months of home monitoring	411 participants recruited. Diagnosis change in 17% and treatment changes in 57%. Mean reduction in exacerbation rate of 0.53 exacerbations per participant. Reductions in unscheduled primary care attendance,	Specialist community clinics improved diagnosis accuracy and adherence to guidelines, reduced exacerbations and was cost saving.

					emergency department visits and hospital admissions,	
Patel, 2024	Cluster RCT	UK	COPD recorded in electronic patient record	Primary care annual COPD review completed by a respiratory specialist.	586 patients randomised. Significant improvement in guideline adherence in the intervention group driven by increased referrals to pulmonary rehabilitation. Higher rates of COPD-related hospitalisations in the intervention group possibly related to differences in data collection methods between groups.	Specialist led care in the community can improve provision of COPD care primarily through increased referral to pulmonary rehabilitation. The effect on emergency care is unclear.
Saini, 2020	Longitudinal matched controlled study	UK	COPD	A consultant led primary care clinic, a nurse led rapid response service, early supported discharge, integrated home oxygen, pulmonary rehabilitation, physiotherapy, counselling and palliative care services.	Non-significant reduction in emergency COPD admissions in the intervention group. Appeared more effective in populations with medium levels of deprivation and in men.	A comprehensive integration of community COPD services showed a small but non-significant reduction in hospital admissions but other health outcomes not collected.
Wolfe, 2023	Cluster RCT	UK	Children (aged <16 years) focusing on children with “tracer” conditions	Local child health clinics for general paediatric problems, delivered by paediatricians and GPs co-consulting and a nurse-led service for children with tracer conditions	No effect at population level on non-elective admissions and no improvement to quality of life scores. Improvements in the	

			(asthma, eczema and constipation).	providing early intervention	quality of care for children with asthma were noted, including better assessment of asthma control and provision of asthma self management plans. Trends to improved cost effectiveness over time.	
Kruis, 2014	Cluster RCT	The Netherlands	COPD according to GOLD criteria	Two day training course for primary care practitioners on incorporating integrated disease management with development of individual practice plans to integrate integrated disease management into daily practice	1086 patients randomised. No difference in quality of life scores at 12 months. Improvement in integration of care and the proportion of physically active patients.	Integrated disease management education for primary care improved integration of care but not patient related outcomes.
Broese, 2023	Cluster RCT	The Netherlands	People with COPD admitted to hospital for an exacerbation considered to have high palliative care needs	An integrated palliative care intervention comprising palliative care conversations tailored to the patient's needs, care coordination and continuity and aftercare if a patient had died	36 of 98 patients in the intervention group received the intervention. No improvement in the primary outcome of quality of life scores at 12 months. Fewer intensive care admissions for COPD and fewer hospitalisations in the intervention group.	Integrated palliative care for COPD may reduce healthcare costs but may not improve quality of life. The COVID-19 pandemic led to suboptimal implementation of the intervention.

RCT: randomised controlled trial. COPD: Chronic Obstruction Pulmonary Disease, GOLD: Global Initiative for Chronic Obstructive Lung Disease.
